# Supplementary material for: ATR regulates neuronal activity by modulating presynaptic firing
Source: Nat Commun. 2021 Jul 1;12:4067. doi: 10.1038/s41467-021-24217-2 (PMC8249387; doi:10.1038/s41467-021-24217-2)
Supplement: Supplementary file 1 — Supplementary Information [file 41467_2021_24217_MOESM1_ESM.pdf]

Supplementary Information

Suppl Figures 1-7

Kirtay et at.

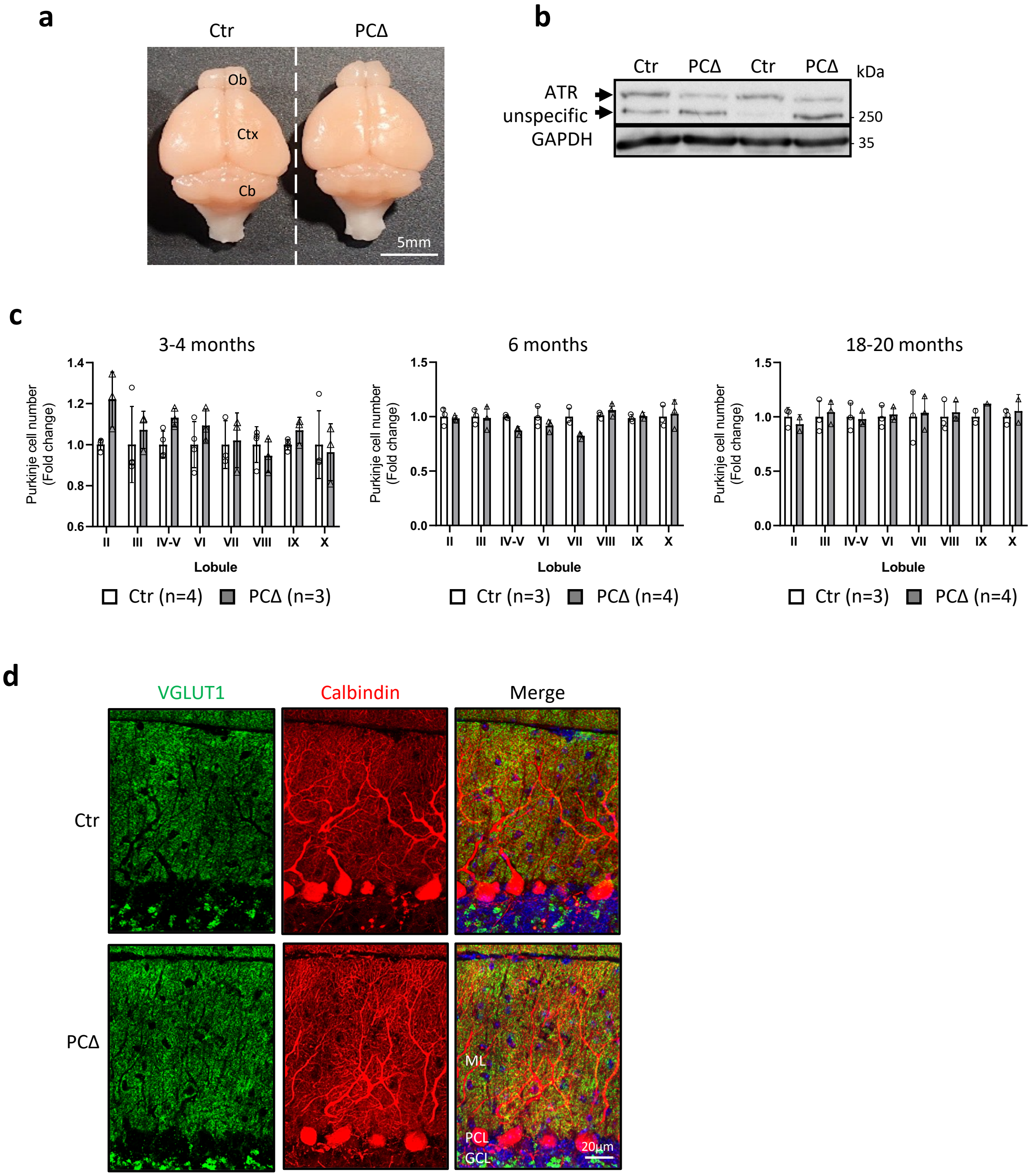

Supplementary Figure 1. ATR-PCΔ brain and cerebellum has normal morphology. (a) Dorsal views of the brains from 3-month-old control (Ctr) and ATR-PCΔ (PCΔ) mice. **(b)** Western blot analysis of cerebellar lysates from 18-month-old ATR-PCΔ mice shows downregulation of ATR. GAPDH was used to control loading. The trace amount of ATR in mutants indicates the presence of non-targeted cells in the cerebellum. The blot is a representative result from 4 mice of each genotype analyzed. **(c)** Quantification of Purkinje cell number in individual cerebella lobules at the indicated age. The data is represented as fold changes compared to the control group. The number of mice (n) analysed of each genotype is indicated. Data are presented as mean values $\pm$ SD. Student's *t*-test (unpaired, two-tailed). No significant difference was detected. **(d)** Immunostaining of cerebellar sections by VGLUT1 and Calbindin. The images are representative from 3 mice of each genotype analyzed. ML: Molecular Layer, PCL: Purkinje Cell Layer, GCL: Granular Cell Layer. Source data are provided as a Source Data file.

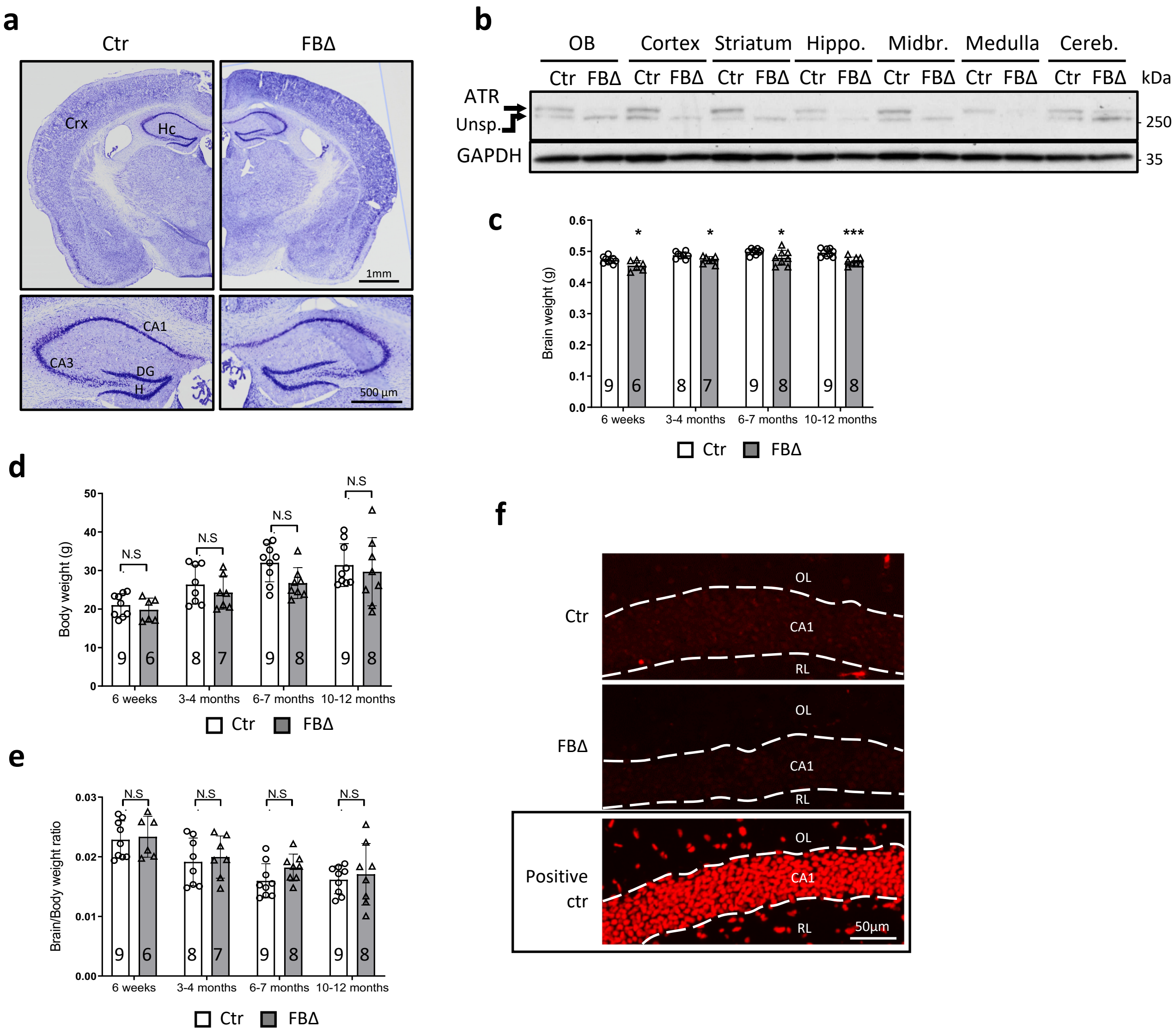

Supplementary Figure 2. ATR deletion in excitatory neurons of the mouse forebrain. (a) Coronal brain sections of 10-month-old control and ATR-FBΔ brains (non-epileptic) were stained with Nissl. The upper panel shows the complete half hemisphere and the lower panel displays the magnified images of the hippocampal regions. The images are representative from 4 mice per genotype analyzed. Ctx: Cortex, Hc: Hippocampus, DG: Dentate Gyrus, CA: Cornu Ammonis, H: Hilus. **(b)** Western blot analysis of the tissue lysates from the different brain regions of 3-month-old control (Ctr) and ATR-FBΔ (FBΔ) mice shows specific deletion of ATR protein in ATR-FBΔ mice. GAPDH used as a loading control. The blot is a representative result from 2-3 mice of each genotype analyzed. Hippo.: Hippocampus, OB: Olfactory Bulb, Midbr.: Midbrain, Cereb.: Cerebellum. **(c-e)** Histograms showing the quantification of brain, body and brain/body weight ratios of control and ATR-FBΔ mice. The number of mice is indicated within each bar. Data are mean values ± SD. Student's *t*-test (unpaired, two-tailed). P-values for **(c):** 6 weeks, p=0.021; 3-4 months, p=0.019; 6-7 months, p=0.041; 10-12 months, p<0.001. *p<0.05, ***p<0.001, N.S., not significant. **(f)** TUNEL staining of brain sections of 3-month-old ATR-FBΔ mice. Images represent the CA1 region of the hippocampus. To control TUNEL staining, DNase treatment was applied. The images are representative from 3-4 mice per genotype analyzed. CA: Cornu Ammonis, OL: Oriens Layer of CA1, RL: Radiatum Layer of CA1. Source data are provided as a Source Data file.

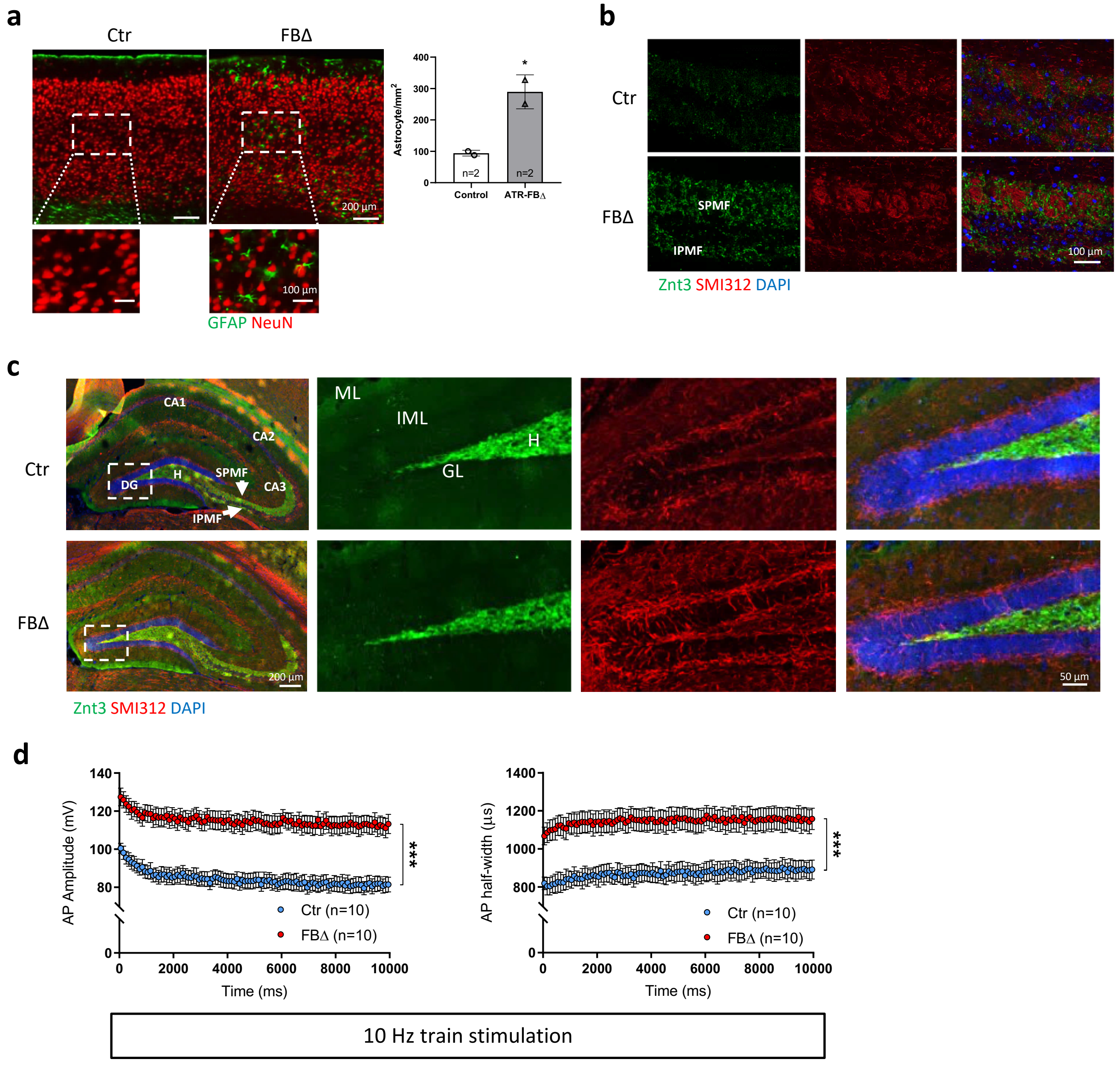

Supplementary Figure 3. ATR deletion in excitatory neurons and epileptic signs of ATR-FBΔ mice. (a) Cortex astrogliosis in ATR-FBΔ mice. Sagittal sections from control and ATR-FBΔ littermates at 10-12 months old after epileptic seizures were stained with GFAP and NeuN to label astrocytes and post-mitotic neurons, respectively. Lower panels show magnifications of the cortex of indicated area. The graph shows the quantification of GFAP-positive cells within the cortical area of 5-6 sections of 2 mice per genotype. The number of mice used is indicated within the bar. Error bars indicate SD. Student's *t*-test. p=0.037. *p<0.05. **(b)** Mossy fiber sprouting in the hippocampus of 10-month-old epileptic ATR-FBΔ mice. Coronal sections of brains were stained with DAPI, ZnT-3 and SMI312 antibodies to label nuclei, mossy fibers and axonal neurofilaments, respectively. The mossy fiber sprouting in the beginning of the CA3 region (ZnT-3 staining) is shown. The images are representative from 3 mice per genotype analyzed. SPMF: Suprapyramidal Mossy Fibers, IPMF: Infrapyramidal Mossy Fibers. **(c)** Analysis of the mossy fiber pathway in 10-month-old non-epileptic mouse hippocampus. Coronal sections of brains were stained with DAPI, ZnT-3 and SMI312 antibodies to label nuclei, mossy fibers and axonal neurofilaments, respectively. White arrows indicate the mossy fiber pathways in IML. Neurofilaments follow the same pattern of ZnT-3 staining. The magnified images of the white rectangles on the DG and CA3 areas are shown in the right panels. The images are representative from 3-4 mice per genotype analyzed. CA: Cornu Ammonis, DG: Dentate Gyrus, SPMF: Suprapyramidal Mossy Fibers, IPMF: Infrapyramidal Mossy Fibers, H: Hilus, GL: Granular Layer, ML: Molecular Layer, IML: Inner Molecular Layer. **(d)** AP amplitudes (left panel) and plot of half-height width of AP (right panel) in whole-cell recorded dentate gyrus granule cells within the 10 Hz train stimulation. Error bars indicate SEM. 2-way ANOVA with Holm-Sidak Post Hoc Analysis. ***p<0.001. Source data are provided as a Source Data file.

a

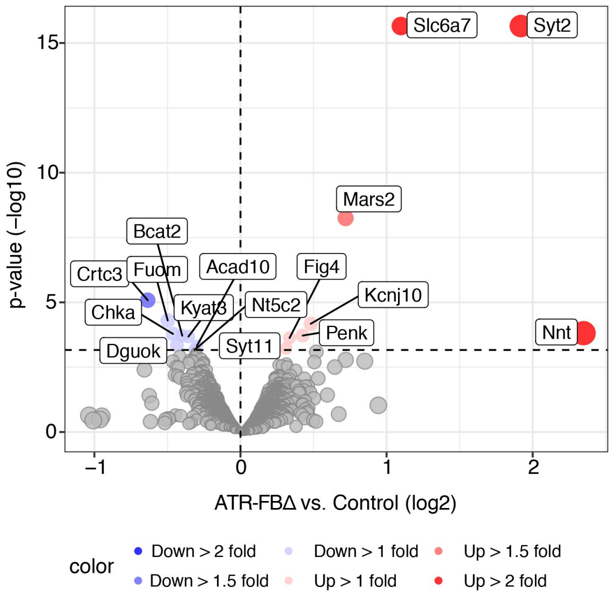

b

Protein Name	Description	Log2 FC	qvalue
SYT2	Synaptotagmin 2	1,9193	6,61E-13
SLC6A7	Sodium-dependent proline transporter (PROT)	1,0980	6,61E-13
MARS2	Methionine--tRNA ligase, mitochondrial	0,7195	1,12E-05
CRTC3	CREB-regulated transcription coactivator 3	-0,6351	0,012
FUCM	Fucose mutarotase	-0,4980	0,054
KCJ10	ATP-sensitive inward rectifier potassium channel 10	0,4809	0,066
NNTM	NAD(P) transhydrogenase, mitochondrial	2,3480	0,100
CHKA	Choline kinase alpha (CK)	-0,4617	0,103
PENK	Proenkephalin-A	0,4255	0,109
BCAT2	Branched-chain-amino-acid aminotransferase	-0,3988	0,109
KYAT3	Kynurenine--oxoglutarate transaminase 3	-0,3562	0,112
FIG4	Polyphosphoinositide phosphatase	0,3360	0,116
DGUOK	Deoxyguanosine kinase, mitochondrial	-0,4344	0,185
ACD10	Acyl-CoA dehydrogenase family member 10	-0,2988	0,196
SYT11	Synaptotagmin 11	0,3120	0,225
5NTC	Cytosolic purine 5’-nucleotidase	-0,3147	0,245

c

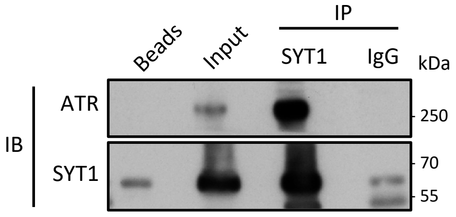

Supplementary Figure 4. Proteome analysis of 3-month-old ATR-FBΔ hippocampus. (a) Volcano plot based on protein quantification by mass spectrometry depicting differentially expressed proteins in ATR-FBΔ hippocampus. The x-axis indicates the log10 p-value (two-tailed) between protein abundance in control (Ctr) and ATR-FBΔ (FBΔ) samples. The horizontal dashed line indicates a q value cut-off of 0.25 (adjusted for multiple testing). Proteins are colored dots as indicated in the figure legend. Significant proteins are highlighted. Four mice of each group were analysed. **(b)** Full list of the proteins that are differentially abundant in ATR-FBΔ hippocampus. The selected SYT2 and SLC6A7 (PROT) proteins are highlighted in green. **(c)** Immunoprecipitation of protein extract from hippocampi using the SYT1 antibody to precipitate ATR. Beads and IgG are controls. Input is 10% of total lysates. The experiment was repeated at least 3 times. Source data are provided as a Source Data file.

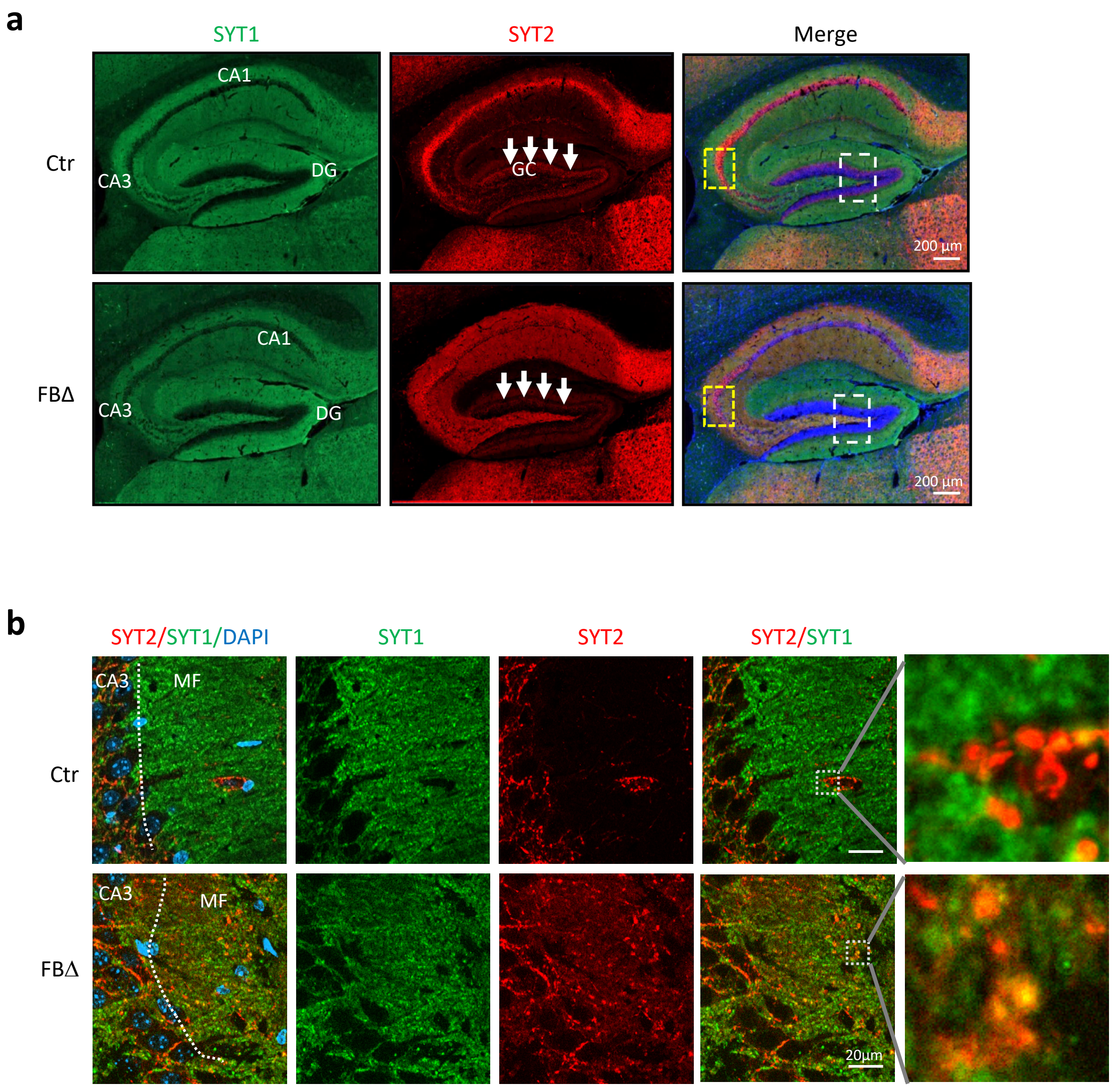

Supplementary Figure 5. Aberrant expression of SYT2 in the synapses of ATR-FBΔ hippocampus. (a) Sagittal sections from 3-month-old control (Ctr) and ATR-FBΔ (FBΔ) mice were stained with DAPI (blue), SYT1 (green) and SYT2 (red). Arrows point to the granule cell layers (GC). Note the absence of STY2 signal in ATR-FBΔ GC layer. Magnification of the white rectangles are shown in Fig. 9a. **(b)** Immunofluorescence analysis of expression pattern of SYT1 and SYT2 in the CA3 area. SYT1 and SYT2 signals are distinctly stained in control mossy fibers, but strongly colocalized in mutant (right zoom-in pictures). The images are representative of 5 mice per genotype analyzed.

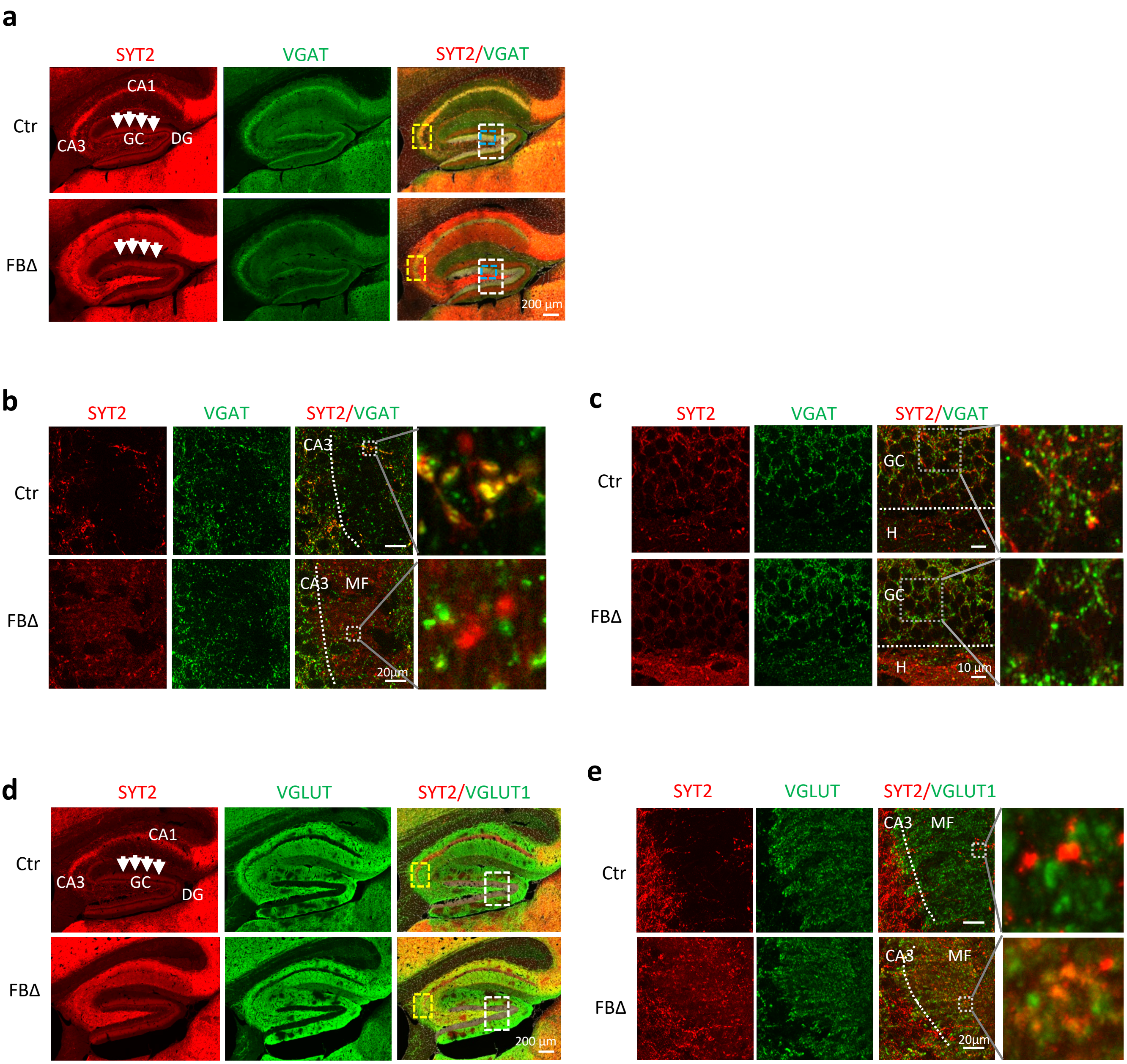

Supplementary Figure 6. Aberrant expression of SYT2 in the excitatory synapses of ATR-FBΔ hippocampus. (a) Sagittal sections from 3-month-old control (Ctr) and ATR-FBΔ (FBΔ) littermates were stained with DAPI (white), SYT2 (red) and VGAT (green, inhibitory synapse marker). Arrows point to the granule cell (GC) layer. Note the absence of STY2 signal in ATR-FBΔ GC layer. Magnification of white rectangles are shown at Fig. 9c. **(b)** Magnification of yellow rectangles in **(a)**. Immunofluorescence analysis of expression pattern of VGAT and SYT2 in the CA3 region. Note a colocalization of VGAT and SYT2 in mossy fibers in controls, which is absent in mutant (zoom-in pictures on right). **(c)** Magnification of blue rectangles in **(a)**. Immunofluorescence analysis of expression pattern of VGAT and SYT2 in the granule cells. Note a colocalization of VGAT and SYT2 in granule cells in controls, which is absent in mutant (zoom-in pictures on right). **(d)** Sagittal sections from 3-month-old control and ATR-FBΔ littermates were stained with DAPI (blue), SYT2 (red) and VGLUT1 (green, excitatory synapse marker). Note the colocalization of STY2 and VLUT1 in ATR-FBΔ hilus. Magnification of white rectangles are shown at Fig. 9e. CA: Cornu Ammonis, DG: Dentate Gyrus. **(e)** Magnification of the yellow rectangles in **(d)**. Immunofluorescence analysis of expression pattern of VLUT and SYT2 in the CA3 area. VGLUT and SYT2 signals are distinctly puncta in control, but strongly colocalized in mutant mossy fibers (right zoom-in pictures). The images are representative of 5 mice per genotype analyzed.

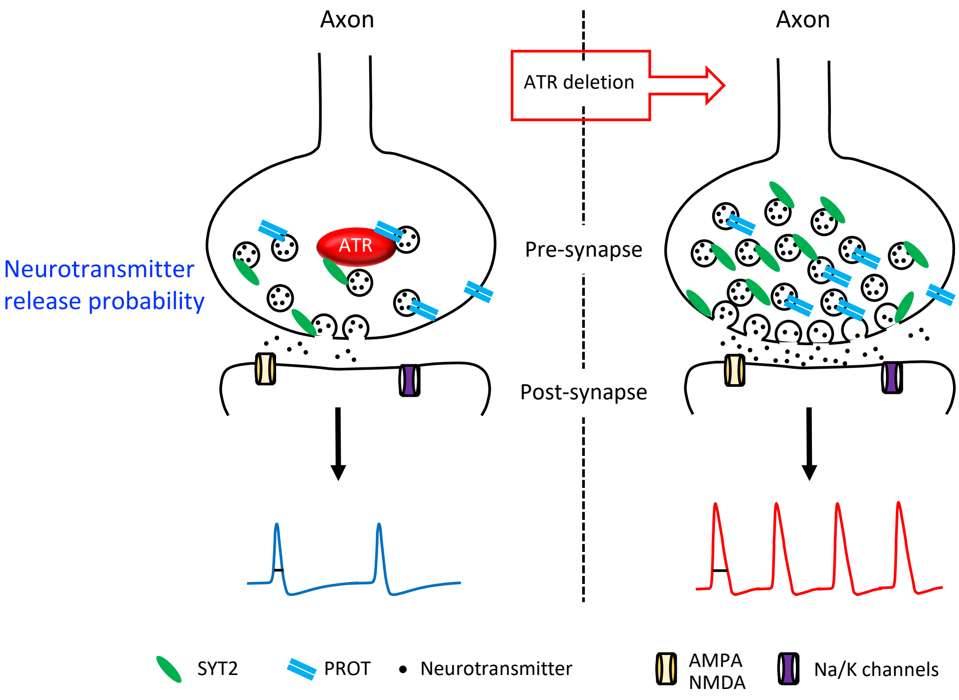

Supplementary Figure 7. Working model. Diagram of ATR-dependent neurotransmission model in neurons. ATR present in the presynaptic compartment and associated with SYT2 and PROT to fine tune the presynaptic homeostasis. Without ATR, Synaptotagmin 2 (SYT2) and L-proline transporter (PROT) are enriched in presynaptic compartment to facilitate transportation and fusion of synaptic vesicles with presynaptic membrane rendering a high probability for the neurotransmitter release and frequent firing.
