# Supplementary material for: ATR regulates neuronal activity by modulating presynaptic firing
Source: Nat Commun. 2021 Jul 1;12:4067. doi: 10.1038/s41467-021-24217-2 (PMC8249387; doi:10.1038/s41467-021-24217-2)
Supplement: Supplementary file 7 — Reporting Summary [file 41467_2021_24217_MOESM7_ESM.pdf]

nature research

Corresponding author(s): Zhao-Qi Wang, PhD
Christian Geis, MD

Last updated by author(s): May 15, 2021

Reporting Summary

Nature Research wishes to improve the reproducibility of the work that we publish. This form provides structure for consistency and transparency in reporting. For further information on Nature Research policies, see our Editorial Policies and the Editorial Policy Checklist.

Statistics

For all statistical analyses, confirm that the following items are present in the figure legend, table legend, main text, or Methods section.

n/a	Confirmed	
☐	☒	The exact sample size (*n*) for each experimental group/condition, given as a discrete number and unit of measurement
☐	☒	A statement on whether measurements were taken from distinct samples or whether the same sample was measured repeatedly
☐	☒	The statistical test(s) used AND whether they are one- or two-sided<br>*Only common tests should be described solely by name; describe more complex techniques in the Methods section.*
☒	☐	A description of all covariates tested
☒	☐	A description of any assumptions or corrections, such as tests of normality and adjustment for multiple comparisons
☐	☒	A full description of the statistical parameters including central tendency (e.g. means) or other basic estimates (e.g. regression coefficient) AND variation (e.g. standard deviation) or associated estimates of uncertainty (e.g. confidence intervals)
☐	☒	For null hypothesis testing, the test statistic (e.g. *F*, *t*, *r*) with confidence intervals, effect sizes, degrees of freedom and *P* value noted<br>*Give P values as exact values whenever suitable.*
☒	☐	For Bayesian analysis, information on the choice of priors and Markov chain Monte Carlo settings
☒	☐	For hierarchical and complex designs, identification of the appropriate level for tests and full reporting of outcomes
☒	☐	Estimates of effect sizes (e.g. Cohen's *d*, Pearson's *r*), indicating how they were calculated

Our web collection on statistics for biologists contains articles on many of the points above.

Software and code

Policy information about availability of computer code

Data collection	For fluorescence microscopy, images were acquired using Zeiss Axio Imager-ApoTome Axiovert 200 ApoTome and ZEN software. Electron micrographs were taken using JEM 1400 electron microscope (JEOL, Japan) and a Orius SC 1000 CCD-camera (GATAN, USA).
Data analysis	Statistical analyses were performed using using GraphPad Prism 8, Sigmaplot 13 and MATLAB 2020. ImageJ 1.53e software was used to perform Western Blot band densitometry, analyses and quantification of fluorescence microscopy images. Analysis of electrophysiology measurements and field potential data were performed with Neuromatic Plugin of IgorPro software (WaveMetrics, Portland, OR, USA) and MATLAB 2020 (MathWorks, Natick, MA, USA), respectively.

For manuscripts utilizing custom algorithms or software that are central to the research but not yet described in published literature, software must be made available to editors and reviewers. We strongly encourage code deposition in a community repository (e.g. GitHub). See the Nature Research guidelines for submitting code & software for further information.

Data

Policy information about availability of data

All manuscripts must include a data availability statement. This statement should provide the following information, where applicable:

- Accession codes, unique identifiers, or web links for publicly available datasets
- A list of figures that have associated raw data
- A description of any restrictions on data availability

The mass spectrometry proteomics data have been deposited to the ProteomeXchange Consortium (http://proteomecentral.proteomexchange.org) (Deutsch et al., 2017; Perez-Riverol et al., 2019; Vizcaino et al., 2014) via the PRIDE partner repository (Vizcaino et al., 2013) with the data set identifier PXD013414 [http://www.ebi.ac.uk/pride/archive/projects/PXD013414] (ATR-FBΔ hippocampus tissue). These data are available for public.
All figures are associated with raw data as Source Data File indicated in the figure legend.

Field-specific reporting

Please select the one below that is the best fit for your research. If you are not sure, read the appropriate sections before making your selection.

- [x] Life sciences
- [ ] Behavioural & social sciences
- [ ] Ecological, evolutionary & environmental sciences

For a reference copy of the document with all sections, see nature.com/documents/nr-reporting-summary-flat.pdf

Life sciences study design

All studies must disclose on these points even when the disclosure is negative.

Sample size	No sample-size calculation was performed. Minimum 3-4 mice were used per analysis which is considered to be acceptable practice in the field, or more as indicated in figure legends. In behavioural tests, sample size was limited by availability of research animals. Sample size was also determined based on the minimum requirement to perform statistical tests.
Data exclusions	No data were excluded from the anayses.
Replication	Described in the figure legends
Randomization	Animals were randomly assigned to experimental groups and group by their genetic identity and age.
Blinding	Investigators were blinded to experimental groups in behavioral, fluorescence image acquisition, TEM, electrophysiology and proteomics studies. Western Blot analyses were not blinded to group the samples in desired order.

Reporting for specific materials, systems and methods

We require information from authors about some types of materials, experimental systems and methods used in many studies. Here, indicate whether each material, system or method listed is relevant to your study. If you are not sure if a list item applies to your research, read the appropriate section before selecting a response.

Materials & experimental systems

n/a	Involved in the study	
[ ]	[x]	Antibodies
[ ]	[x]	Eukaryotic cell lines
[x]	[ ]	Palaeontology and archaeology
[ ]	[x]	Animals and other organisms
[x]	[ ]	Human research participants
[x]	[ ]	Clinical data
[x]	[ ]	Dual use research of concern

Methods

n/a	Involved in the study	
[x]	[ ]	ChIP-seq
[x]	[ ]	Flow cytometry
[x]	[ ]	MRI-based neuroimaging

Antibodies

Antibodies used	Western Blot: ATR 1:250 (SantaCruz, #sc-515173), GAPDH 1:5000 (Sigma-Aldrich,#G8795), ATM 1:1000 (Novus Biologicals, #NB100-309), PAR 1:1000 (Trevigen, #4336-BPC-100), PARP1 1:1000 (SantaCruz, #sc-1561), Chk1 1:1000 (Bethyl Laboratories,#A300-162A), phospho-Chk1 1:1000 (Bethyl Laboratories, #A300-163A), γH2AX S139 1:1000 (Upstate, #05-636), Syt1 1:1000 (Synaptic Systems, #105011), Syt2 1:1000 (Synaptic Systems, #105123), Slc6a7 1:400 (GeneTex, #GTX65943), NR2B 1:1000 (Millipore, #06-600), GluR1 1:1000 (SantaCruz, #sc-13152), Kv1.1 1:200 (Alomone, #APC-009), GAD67 1:1000 (Abcam, #ab26116), phospho-(S/T) ATM/ATR substrate 1:1000 (Cell Signaling, #2851S), tdTomato 1:1000 (SICGEN, #AB8181-200), H3 1:1000 (Abcam #ab1791).<br><br>Immunofluorescence: calbindin D28K 1:1000 (SantaCruz, #7691), GFAP 1:4000 (Dako, #Z0334), NeuN 1:4000 (Millipore, #MAB377), SMI312 1:4000 (Covance, #SMI312-R), Znt3 1:200 (Synaptic Systems, #197002), Syt1 1:200 (Synaptic Systems, #105011), Syt2 1:200 (Synaptic Systems, #105123), VGAT 1:200 (Synaptic Systems, #131003), VGLUT1 1:200 (Synaptic Systems, #135303), Streptavidin-Cy3 1:800 (Sigma, #S6402-1ml), anti-mouse Cy3 1:200 (Sigma, #C2181-1Ml), anti-rabbit Cy3 1:200 (Sigma, #C2306-1Ml), anti-rabbit Cy2 1:200 (Jackson Immuno Research, #711-225-152), anti-rabbit Cy5 1:200 (Jackson Immuno Research, #711-175-152), Alexa Fluor 488 donkey anti-Mouse igg (H+L) 1:200 (Invitrogen, #A21202)
Validation	All antibodies were obtained commercially and validation information can be obtained from the manufacturers using the antibody catalog numbers provided in the Methods section and also this reporting summary.

Eukaryotic cell lines

Policy information about cell lines

Cell line source(s)	Neuroblastoma cells, ATCC® CCL-131
Authentication	None of the cell lines were authenticated.
Mycoplasma contamination	All the cell lines were tested negative for mycoplasma contamination.
Commonly misidentified lines (See ICLAC register)	No commonly misindentified lines were used in this study.

Animals and other organisms

Policy information about studies involving animals; ARRIVE guidelines recommended for reporting animal research

Laboratory animals	mouse, C57BL/6, 129Sv, males and females. Age are defined in the text, figures, and figure legends. All animal experiments and breeding were conducted according to the German animal welfare legislation and approved by the Thüringer Landesverwaltungsamt (animal license nr.: 03-042/16).
Wild animals	No wild animals were used in this study.
Field-collected samples	No field collected samples were used in this study.
Ethics oversight	*Identify the organization(s) that approved or provided guidance on the study protocol, OR state that no ethical approval or guidance was required and explain why not.*

Note that full information on the approval of the study protocol must also be provided in the manuscript.
