# Supplementary material for: ATR regulates neuronal activity by modulating presynaptic firing
Source: Nat Commun. 2021 Jul 1;12:4067. doi: 10.1038/s41467-021-24217-2 (PMC8249387; doi:10.1038/s41467-021-24217-2)
Supplement: Supplementary file 8 — Description of Additional Supplementary Files [file 41467_2021_24217_MOESM8_ESM.pdf]

**Title:** Supplementary Movie 1.
**Description:** Crossbeam test of control animals.

**Title:** Supplementary Movie 2.
**Description:** Crossbeam test of ATR-PCΔ mice animals. Mutant mice took long time to complete the task and with many foot slips.

**Title:** Supplementary Movie 3.
**Description:** Seizure attack in ATR-FBΔ mice animals (short epileptic video). Sudden onset of tonic-clonic generalized seizure with myoclonic jerks of all limbs followed by a short tonic phase.

**Title:** Supplementary Movie 4.
**Description:** Seizure attack in ATR-FBΔ mice animals. Generalized seizure with dystonic movement of the hindlimbs.

**Title:** Supplementary Movie 5.
**Description:** Seizure attack in ATR-FBΔ mice animals. Generalized tonicclonic seizure with hyperactive phase followed by postictal slowing
